# Evaluation of cardiotoxicity of anthracycline‐containing chemotherapy regimens in patients with bone and soft tissue sarcomas: A study of the FDA adverse event reporting system joint single‐center real‐world experience

**DOI:** 10.1002/cam4.6730

**Published:** 2023-12-06

**Authors:** Zeming Mo, Yaotiao Deng, Yiwen Bao, Jie Liu, Yu Jiang

**Affiliations:** ^1^ Division of Medical Oncology, Cancer Center, West China Hospital Sichuan University Chengdu China; ^2^ Department of Oncology The People's Hospital of Qiannan Duyun Guizhou China

**Keywords:** ADM, anthracyclines, bone and soft tissue sarcomas, cardiotoxicity, FDA adverse event reporting system

## Abstract

**Objectives:**

To assess the occurrence of cardiotoxicity in patients with tumors receiving anthracycline‐based chemotherapy, especially for sarcomas.

**Methods:**

This study summarized the types and frequency of adverse events (AEs) for three anthracyclines from the FDA adverse event reporting system (FAERS) database. FAERS data from January 2004 to June 2022 were collected and analyzed. Disproportionality analyses, logistic regression, and descriptive analysis were used to compare the differences in cardiac disorders. A retrospective cohort study was conducted in a single center between December 2008 and May 2022. Our hospital‐treated patients with bone and soft tissue sarcomas (BSTSs) with anthracycline‐containing chemotherapy were analyzed. Serum markers, echocardiography, and electrocardiography have been used to evaluate cardiotoxic events.

**Results:**

One hundred thousand and seventy‐five AE reports were obtained for doxorubicin (ADM), epirubicin (EPI), and liposome doxorubicin (L‐ADM) from the FAERS database. ADM (OR = 3.1, *p* < 0.001), EPI (OR = 1.5, *p* < 0.001), and sarcomas (OR = 1.8, *p* < 0.001) may increase the probability of cardiac disorders. Cardiac failure, cardiotoxicity, and cardiomyopathy were anthracyclines' top 3 frequent AEs. Among patients receiving ADM‐containing therapy, those with ADM applied at doses ≥75 mg/m^2^/cycle were more likely to develop cardiac disorders than the other subgroups (OR = 3.5, *p* < 0.001). Patients younger than 18 are more likely to benefit from dexrazoxane prevention of cardiac failure. Six hundred and eighty‐three patients with BSTSs receiving anthracycline‐based chemotherapy were analyzed in our center. Patients receiving ADM‐containing chemotherapy were likelier to experience abnormalities in serum troponin‐T and left ventricular ejection fraction (*p* < 0.05). 2.0% (6/300) of patients receiving ADM‐containing chemotherapy required adjustment of the chemotherapy regimen because of cardiotoxicity, whereas none were in the EPI or L‐ADM groups.

**Conclusions and Relevance:**

Among patients receiving anthracycline‐containing therapy, patients with BSTSs were more likely to develop cardiac disorders than other tumors. In addition, patients with BSTSs receiving ADM chemotherapy had a higher likelihood of cardiotoxic events than those receiving EPI or L‐ADM.

## 1 INTRODUCTION

Anthracyclines have been widely used in various solid tumors and hematologic malignancies since the 1950s and still occupy a significant place in standard chemotherapy regimens.^1^ Anthracyclines remain among the most effective chemotherapeutic drugs for treating many tumors, including sarcoma, lymphoma, leukemia, and breast cancer (BC).^2^ Currently, anthracyclines mainly include doxorubicin (ADM), epirubicin (EPI), daunorubicin, amrubicin, and idarubicin. In addition, it has been 28 years since Doxil®, the first antitumor liposome doxorubicin (L‐ADM) approved by the FDA, was used.^3^ ADM, EPI, and L‐ADM are currently approved for use in both solid tumors and hematologic malignancies. However, daunorubicin and idarubicin are only approved for hematologic malignancies (https://www.fda.gov/drugs). Amrubicin is only approved for lung cancer.^4^ Anthracyclines damage cardiomyocytes by inserting DNA directly, inhibiting topoisomerase, and disrupting organelle structure by generating free radical species.^5^ For example, heart failure occurs in 4.4% of childhood cancer survivors (CCSs), and severe or life‐threatening heart failure is higher in recent periods.^6^ Once diagnosed with congestive heart failure, CCS has a 5‐year survival rate of less than 50%.^2^ In addition, asymptomatic left ventricular (LV) dysfunction incidence was significantly higher in CCS patients treated with anthracyclines than in those with heart failure.^7^ Cardiotoxicity is also alarming for adult cancer patients.^8^, ^9^ Therefore, the risk of anthracycline‐induced cardiotoxicity is always considered when formulating antineoplastic chemotherapy.

Whether different anthracycline derivatives induce different grades of cardiotoxicity risk has not been determined.^10^ Some studies have shown that there is no difference in the risk of the occurrence of clinical heart failure between ADM and EPI.^11^, ^12^ However, other studies suggested that chemotherapy regimens containing EPI had less cardiotoxicity than those containing ADM.^13^, ^14^ In addition, some studies have suggested that liposomal anthracyclines may reduce cardiotoxicity while ensuring antitumor efficacy compared to ADM or EPI in treating BC.^15^, ^16^, ^17^ However, other findings have questioned the conclusion that L‐ADM chemotherapy regimens can reduce cardiotoxicity in the treatment of lymphoma.^18^, ^19^ Therefore, the cardiotoxicity of chemotherapy regimens containing ADM, EPI, or L‐ADM may not be the same in different tumors.

In most cases, the first‐line treatment for advanced bone and soft tissue sarcomas (BSTSs) is doxorubicin‐based chemotherapy.^20^, ^21^, ^22^ Even anthracyclines are widely used in the neoadjuvant and adjuvant treatment of sarcomas. One point of note is that patients with multiple types of sarcomas often receive high doses of anthracycline per cycle (both ADM and EPI: ≥75 mg/m^2^/cycle) in chemotherapy regimens.^23^, ^24^ The FDA adverse event reporting system (FAERS) database can provide detailed information on cardiotoxic adverse events (AEs) associated with anticancer therapy.^25^, ^26^ However, few studies have focused on the comparative analysis of differences in the type and frequency of cardiac disorders among different cancer types with chemotherapy regimens containing ADM, EPI, or L‐ADM. In particular, comparing the likelihood of cardiotoxicity occurring between BSTSs and other tumors is essential.

In addition, dexrazoxane is uniquely approved by the FDA for managing and treating anthracycline‐induced cardiotoxicity and extravasation injuries.^27^ Therefore, it is necessary to evaluate the cardioprotective effect of dexrazoxane in patients with tumors treated with anthracyclines based on the FAERS database.

Most cardiotoxicity due to chemotherapy regimens containing anthracyclines occurs during the first year of treatment, and partial or complete recovery can be achieved in more than 80% of patients with early identification and intervention.^28^ Therefore, it is critical to identify the onset of cardiotoxicity early in chemotherapy effectively. However, the diagnosis of asymptomatic LV dysfunction is difficult. This study aims are to compare the likelihood of cardiotoxicity occurring between sarcomas and other tumors receiving anthracycline‐based chemotherapy based on the FAERS database and provide data on the real‐life cardiotoxicity of anthracycline‐containing chemotherapy regimens in patients with multiple types of sarcomas from a single‐center study. We sought to analyze whether there are differences in the incidence of early cardiotoxicity induced by chemotherapy regimens containing different anthracyclines. Abnormalities in biomarkers were also assessed.

## 2 METHOD

### 2.1 FAERS database data collection and cleaning

The retrospective pharmacovigilance study data were obtained from the FAERS database (https://fis.fda.gov/extensions/FPD‐QDE‐FAERS/FPD‐QDE‐FAERS.html). The FAERS database was searched (1 January 2004–30 June 2022) for AEs data of anthracyclines commonly used to treat hematologic malignancies and solid tumors (ADM, EPI, and L‐ADM). More than 99% of daunorubicin and idarubicin AEs in the FAERS database were related to the treatment of hematologic malignancies. In addition, amrubicin‐related AEs remained unrecorded in the FAERS database. Therefore, daunorubicin, idarubicin, and amrubicin were excluded. We only selected reported events for anthracyclines judged to be primary suspect (PS) and second suspect (SS) in “ROLE_COD”. Only cases with the latest uploaded FDA_DT were selected if there was a duplicate CASEID and PRIMARYID prior to analysis. Based on the Medical Dictionary for Regulatory Activities (MedDRA), the preferred term (PT) was used to standardize data, and systematic organic classification (SOC) was utilized to classify AEs into various systems. The data cleaning process relied on SAS statistical software (version 9.4; SAS Institute, Cary, NC, USA). After data cleaning, 77,801, 13,172, and 9102 patients were eventually found to have received chemotherapy containing ADM, EPI, and L‐ADM, respectively.

### 2.2 Screening of patients who received anthracycline therapy along with dexrazoxane in the FAERS database

To evaluate the effectiveness of dexrazoxane in mitigating cardiotoxicity of anthracyclines‐containing chemotherapy, those patients who had both anthracyclines (ADM, EPI, or L‐ADM) and dexrazoxane/ZINECARD in the “DRUGNAME” were selected as the “Dexrazoxane group”, and those who had only anthracyclines were selected as the “None‐dexrazoxane group”, based on the FAERS database.

### 2.3 Screening of patients in our center

This retrospective single‐center study conformed to the principles of the Declaration of Helsinki. Approval was granted by the Ethics Committee of West China Hospital of Sichuan University, China (No. 627 in 2020). We retrospectively collected data from patients treated with at least one cycle of anthracycline‐containing chemotherapy in our hospital between December 2008 and May 2022, including adjuvant, first‐line, or multiline therapy. All patients were pathologically diagnosed with BSTSs. Similarly, patients with concomitant malignancies of other types were also excluded. Patients received intravenous infusion of standard‐dose anthracycline‐containing chemotherapy for 4–6 cycles or until disease progression or unacceptable toxic events. After screening and validation, 300, 341, and 112 patients were eventually found to have received chemotherapy containing ADM, EPI, and L‐ADM, respectively, in our center.

### 2.4 Outcomes

In the single‐center retrospective study, the primary endpoint was cardiac safety of chemotherapy assessed by incidence of New York Heart Association (NYHA) class III and IV heart failure or left ventricular ejection fraction (LVEF) declines (10% from baseline and to a value of <50% [symptomatic and asymptomatic]) by echocardiography.^29^ LVEF assessment was performed at screening/baseline before the first cycle of anthracycline‐containing chemotherapy and reassessed every ≥2 cycles of chemotherapy. Secondary endpoints were the serum levels of troponin‐T (TPN‐T) and N‐terminal BNP (NT‐ProBNP) and electrocardiographic abnormalities.

### 2.5 Statistical analysis

#### 2.5.1 Disproportionality analysis

The proportional reporting ratio (PRR) method and comprehensive standard method (MHRA) were used to determine the safety signals of the drugs. Calculations are as described earlier.^30^ Those PTs that were judged to have positive signals were considered to be possible target drug‐related AEs. The positive signal determination criteria for MHRA were as follows: the number of cases of AEs was greater than three, PRR ≥2, and the lower limit of the chi‐square value ≥4.

#### 2.5.2 Logistic regression

Univariate or multivariate logistic regression was performed and adjusted OR values were calculated through the R statistical package broom (version 1.0.4).^31^ The MHRA signal is the dependent variable, where “1” represents a positive signal and “0” represents a negative signal. Covariates included drug types, age, sex, cancer type, etc. An odds ratio (OR) >1 suggests that exposure can promote outcomes.

#### 2.5.3 Data visualization

Statistical analyses and drawings were performed using RStudio software (2023.03.0 + 386) based on the R program (https://www.r‐project.org/, version: 4.2.3) and GraphPad Prism 9. Comparisons between groups were carried out with *t*‐tests and Wilcoxon rank‐sum tests. The Cox regression hazard model was conducted using the R statistical packages “survival” and “survminer”.^32^ R packages including ggplot2 (version 3.4.1), ggforestplot (version 3.4.1), forestplot (version 3.4.1) and ComplexHeatmap (version 3.4.1) were employed for data visualization. Statistical analyses were also performed using GraphPad Prism 9. *p*‐values <0.05 were considered statistically significant.

## 3 RESULTS

### 3.1 Basic clinical characteristics of patients treated with anthracycline‐containing chemotherapy from the FAERS database

**FIGURE 1:**
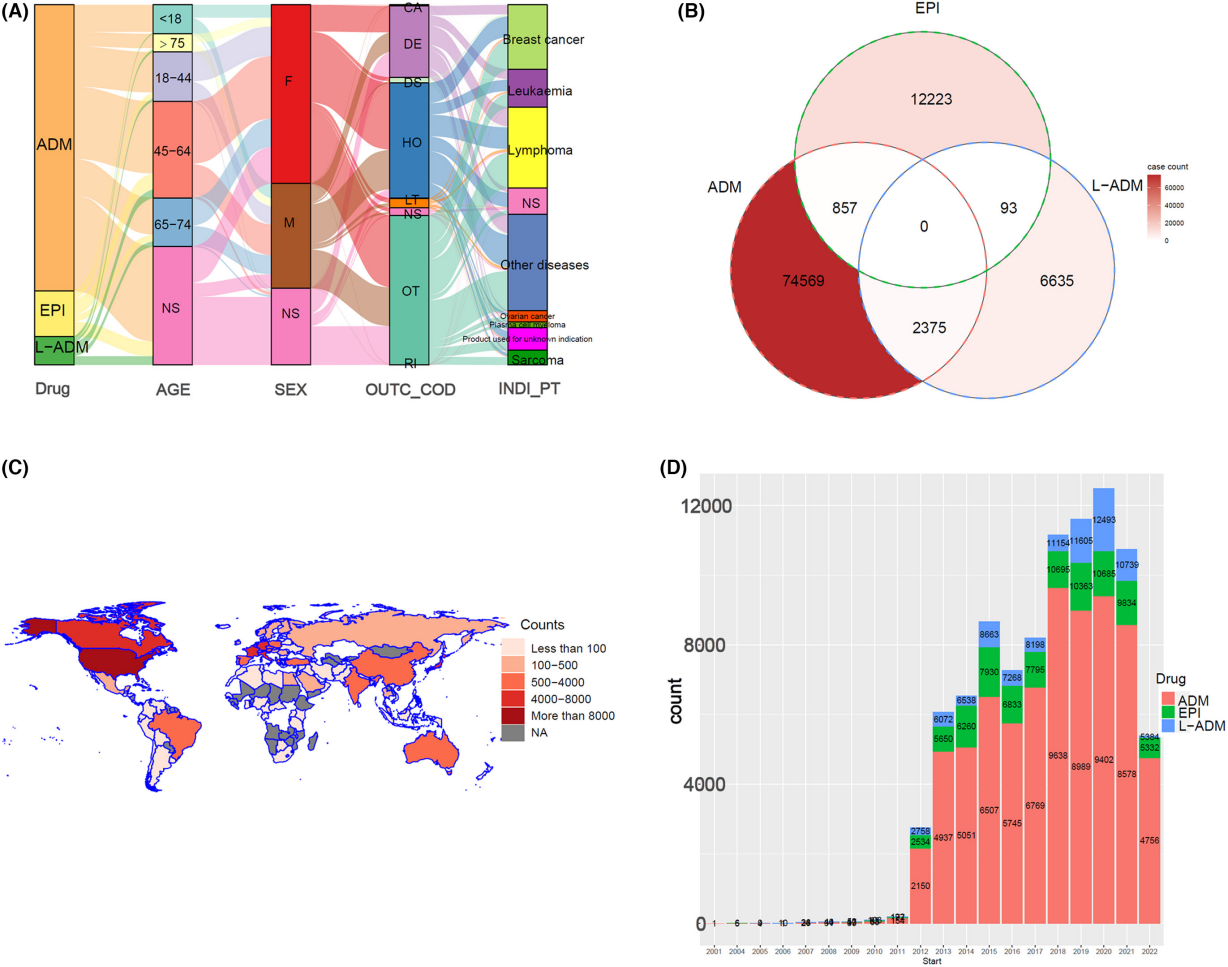
Distribution of essential clinical characteristics of patients receiving anthracycline‐containing chemotherapy from FAERS database. (A) Mulberry map of clinical characteristics of patients with doxorubicin (ADM), epirubicin (EPI), and liposomal doxorubicin (L‐ADM). Data were collected from drugs, age, sex, outcome (OUTC_COD), and cancer types (INDI_PT). (B) Overlapping Wayne plots of individual identities of patients treated with three medications based on PRIMARYID values. (C) Distribution of countries and number of cases of patients included in all reported adverse events (AEs) for the three drugs. (D) The application frequency of anthracyclines was counted. NS indicates those not explicitly stated in the factor.

Data originating from FAERS were de‐duplicated and cleaned, and the final number of patients included in the analysis who had been treated with ADM‐containing (77,801 cases), EPI‐containing (77,801 cases), or L‐ADM‐containing (9102 cases) regimens all exceeded 9000. As shown in Figure 1A, except for data not explicitly labeled with age (NS), the number of patients in the 45–64 age range was the highest percentage (38.0%). This is followed by the 18–44 years old range (20.7%) and the 65–74 years old range (19.7%), which have very similar shares. In the gender statistics, the percentage of women (59.9%) was higher than that of men (40.1%). The percentage of women in EPI‐containing chemotherapy regimens was as high as 84.7%, which may be related to the fact that EPIs are mainly used in BC treatments (59.0%) (Table 1). In contrast, ADM has a relatively small difference in the number of applications for men and women patients. In the distribution of outcomes (OUTC_COD), Other Serious (OT), Hospitalization ‐ Initial or Prolonged (HO), and Death (DE) were the top three in frequency. Lymphoma, BC, and leukemia are the top three most frequent malignancies. In the subgroup analysis, ADM was mainly used in lymphoma (30%) and leukemia (15%), while L‐ADM was mainly used in ovarian cancer (20%), lymphoma (19%), and BC (14%). In addition, we noted that some patients were treated with two of the three drugs sequentially (Figure 1B). The highest percentage was among those L‐ADM patients who had also been treated with ADM (26.4%). The United States (16,970 cases), France (7648 cases), Italy (5568 cases), and Canada (5547 cases) ranked in the top four countries in terms of the total number of reported AEs suspected to be related to anthracyclines (Figure 1C). The number of reported cases showed a relatively significant increase since 2012 until it peaked in 2020 at 12,493 (Figure 1D).

**TABLE 1:** Clinical characteristics of patients treated with anthracycline‐containing regimens from the FAERS database.

Characteristic	ADM, *N* = 77,801^a^	EPI, *N* = 13,172^a^	L‐ADM, *N* = 9102^a^	*p*‐Value^b^
Age				
<18	8379 (11%)	259 (2.0%)	323 (3.5%)	<0.001
≥75	4151 (5.3%)	396 (3.0%)	755 (8.3%)	
18–44	11,254 (14%)	1741 (13%)	907 (10.0%)	
45–64	18,450 (24%)	4649 (35%)	2717 (30%)	
65–74	9730 (13%)	1873 (14%)	1638 (18%)	
NS	25,837 (33%)	4254 (32%)	2762 (30%)	
Gender				
Female	31,911 (41%)	9286 (70%)	5635 (62%)	<0.001
Male	27,474 (35%)	1680 (13%)	2161 (24%)	
NS	18,416 (24%)	2206 (17%)	1306 (14%)	
OUTC_COD				
CA	234 (0.3%)	18 (0.1%)	0 (0%)	<0.001
DE	16,731 (22%)	2191 (17%)	1447 (16%)	
DS	817 (1.1%)	390 (3.0%)	117 (1.3%)	
HO	24,867 (32%)	4211 (32%)	2837 (31%)	
LT	2195 (2.8%)	300 (2.3%)	210 (2.3%)	
NS	1700 (2.2%)	91 (0.7%)	557 (6.1%)	
OT	31,211 (40%)	5958 (45%)	3921 (43%)	
RI	46 (<0.1%)	13 (<0.1%)	13 (0.1%)	
INDI_PT				
Breast cancer	6248 (8.0%)	7745 (59%)	1262 (14%)	<0.001
Leukemia	11,730 (15%)	130 (1.0%)	214 (2.4%)	
Lymphoma	23,034 (30%)	656 (5.0%)	1767 (19%)	
NS	5892 (7.6%)	1181 (9.0%)	892 (9.8%)	
Other diseases	21,138 (27%)	3056 (23.2%)	2046 (22%)	
Ovarian cancer	1523 (2.0%)	94 (0.7%)	1812 (20%)	
Unknown indication	4616 (5.9%)	0 (0%)	710 (7.8%)	
Sarcoma	3620 (4.7%)	310 (2.4%)	399 (4.4%)	

*Note*: NS indicates that there is no clearly labeled information.

^a^ *n* (%).

^b^ Pearson's chi‐squared test.

### 3.2 ADM, EPI, and sarcomas may increase the probability of cardiac disorders

**FIGURE 2:**
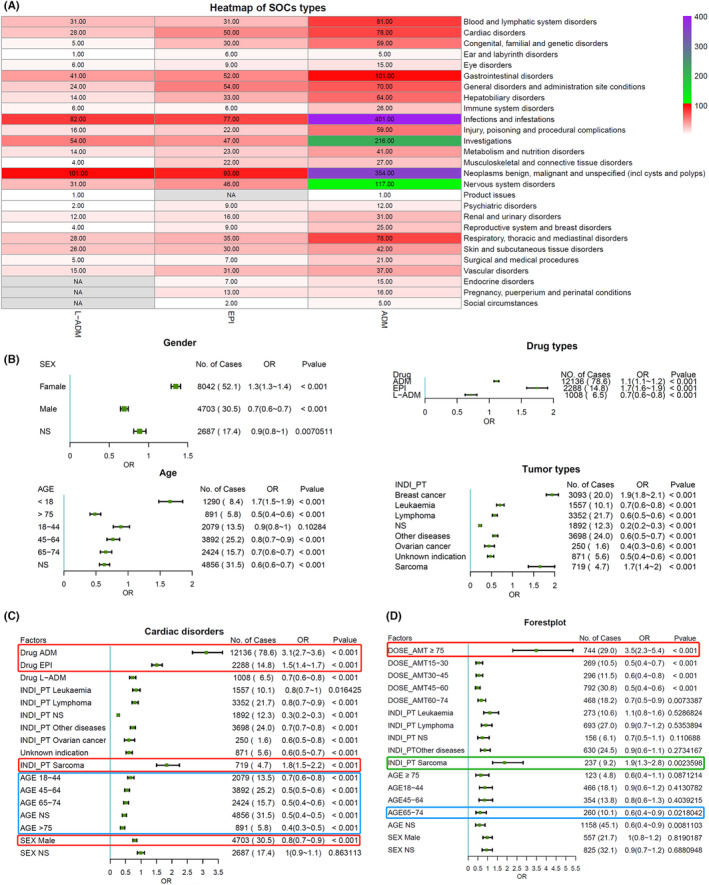
Distribution of types of cardiac disorders and comparison of the likelihood of adverse events (AEs) between different subgroups. (A) Heat map distribution of the types of suspected anthracycline‐related AEs in the different system organ classes (SOCs). (B) Univariate logistic regression analyses were performed with the MHRA signal as the dependent variable. (C) Multivariate logistic regression analyses were performed with the MHRA signal as the dependent variable. (D) AEs related to cardiac disorders of ADM were included for analysis, and multivariate logistic regression analyses were performed.

AEs originating from the FAERS database were standardized to be classified into each SOC based on MedDRA rules. Subsequently, the number of PT types of the three anthracyclines was counted in all SOC categories (Figure 2A). The types of PTs in “infections,” “neoplasms benign, malignant and unspecified,” and “investigations” were the top three most frequent. Significantly higher frequencies of infection‐related AEs, such as sepsis, septic shock, infection, and respiratory failure, occurred in those patients who died relative to those who survived (Figure S1). These results suggest that we should pay attention to the prevention and treatment of sepsis when applying anthracycline‐based chemotherapy.

Otherwise, the PT type of ADM‐related cardiac disorders (78 types) was higher than that of EPI (50 types) and L‐ADM (28 types). Univariate and multivariate logistic regression analyses were applied to attenuate the interference of statistical results due to differences in the total number of AEs reported cases between drugs. The results of univariate regression analyses suggest a higher likelihood of cardiac disorders in women relative to men, EPI and ADM relative to L‐ADM, less than 18 years of age relative to other age groups, and BC and sarcoma relative to other tumors (Figure 2B). The results of the multivariate regression analysis reaffirmed that factors such as ADM, EPI, BC, sarcoma, and being younger than 18 years of age may be more likely to produce cardiac disorders (Figure 2C). The ORs for ADM (OR = 3.1, *p* < 0.001) and sarcoma (OR = 1.8, *p* < 0.001) were the top two in the multivariate analysis, while 83.62% of patients with sarcoma had been treated with ADM (Table 1). Therefore, we hypothesized that the high probability of cardiac disorders in patients with sarcoma may correlate with ADM use. As previously mentioned, sarcoma patients often receive ≥75 mg/m^2^/cycle of ADM for systemic therapy, and the cardiotoxicity of ADM has a significant dose‐cumulative effect. We categorized the per‐cycle dose of ADM (units: mg/m^2^/cycle) into five intervals, including 15–30, 30–45, 45–60, 60–75, and ≥75, and performed multivariate analyses with the MHRA signals as the independent variables. The results suggest a significantly higher likelihood of cardiac disorders in the group ≥75 mg/ m^2^/cycle (OR = 3.5, *p* < 0.001) (Figure 2D). In addition, patients with sarcomas receiving ADM‐containing chemotherapy regimens were more likely to develop cardiac disorders than other tumors. Moreover, 72% of cardiac disorder‐related AEs in sarcoma patients were pooled in the ≥75 mg subgroup, twice as high as second‐ranked leukemia (Table 2). Therefore, high doses of ADM are likely to increase cardiotoxicity in sarcoma patients.

**TABLE 2:** Clinical characteristics of overall patients with cardiac disorders in ADM group.

Characteristic	Breast cancer, *N* = 580^a^	Leukemia, *N* = 273^a^	Lymphoma, *N* = 693^a^	NS, *N* = 156^a^	Other diseases, *N* = 630^a^	Sarcoma, *N* = 237^a^	*p*‐Value^b^
Age							
<18	2 (0.3%)	58 (21%)	11 (1.6%)	25 (16%)	61 (9.7%)	51 (22%)	<0.001
≥75	3 (0.5%)	2 (0.7%)	82 (12%)	5 (3.2%)	22 (3.5%)	9 (3.8%)	
18–44	130 (22%)	61 (22%)	74 (11%)	24 (15%)	113 (18%)	64 (27%)	
45–64	92 (16%)	26 (9.5%)	54 (7.8%)	68 (44%)	96 (15%)	18 (7.6%)	
65–74	60 (10%)	22 (8.1%)	84 (12%)	11 (7.1%)	69 (11%)	14 (5.9%)	
NS	293 (51%)	104 (38%)	388 (56%)	23 (15%)	269 (43%)	81 (34%)	
Gender							
Female	504 (87%)	111 (41%)	145 (21%)	92 (59%)	231 (37%)	104 (44%)	<0.001
Male	4 (0.7%)	66 (24%)	186 (27%)	47 (30%)	201 (32%)	53 (22%)	
NS	72 (12%)	96 (35%)	362 (52%)	17 (11%)	198 (31%)	80 (34%)	
DOSE_AMT							
>75	113 (19%)	99 (36%)	143 (21%)	42 (27%)	177 (28%)	170 (72%)	<0.001
15–30	3 (0.5%)	10 (3.7%)	71 (10%)	30 (19%)	139 (22%)	16 (6.8%)	
30–45	18 (3.1%)	44 (16%)	53 (7.6%)	7 (4.5%)	153 (24%)	21 (8.9%)	
45–60	142 (24%)	73 (27%)	422 (61%)	28 (18%)	122 (19%)	5 (2.1%)	
60–74	304 (52%)	47 (17%)	4 (0.6%)	49 (31%)	39 (6.2%)	25 (11%)	

*Note*: NS indicates that there is no clearly labeled information.

^a^ *n* (%).

^b^ Pearson's chi‐squared test.

### 3.3 Cardiac failure, cardiotoxicity, and cardiomyopathy were the top three frequent AEs for all three anthracyclines

**FIGURE 3:**
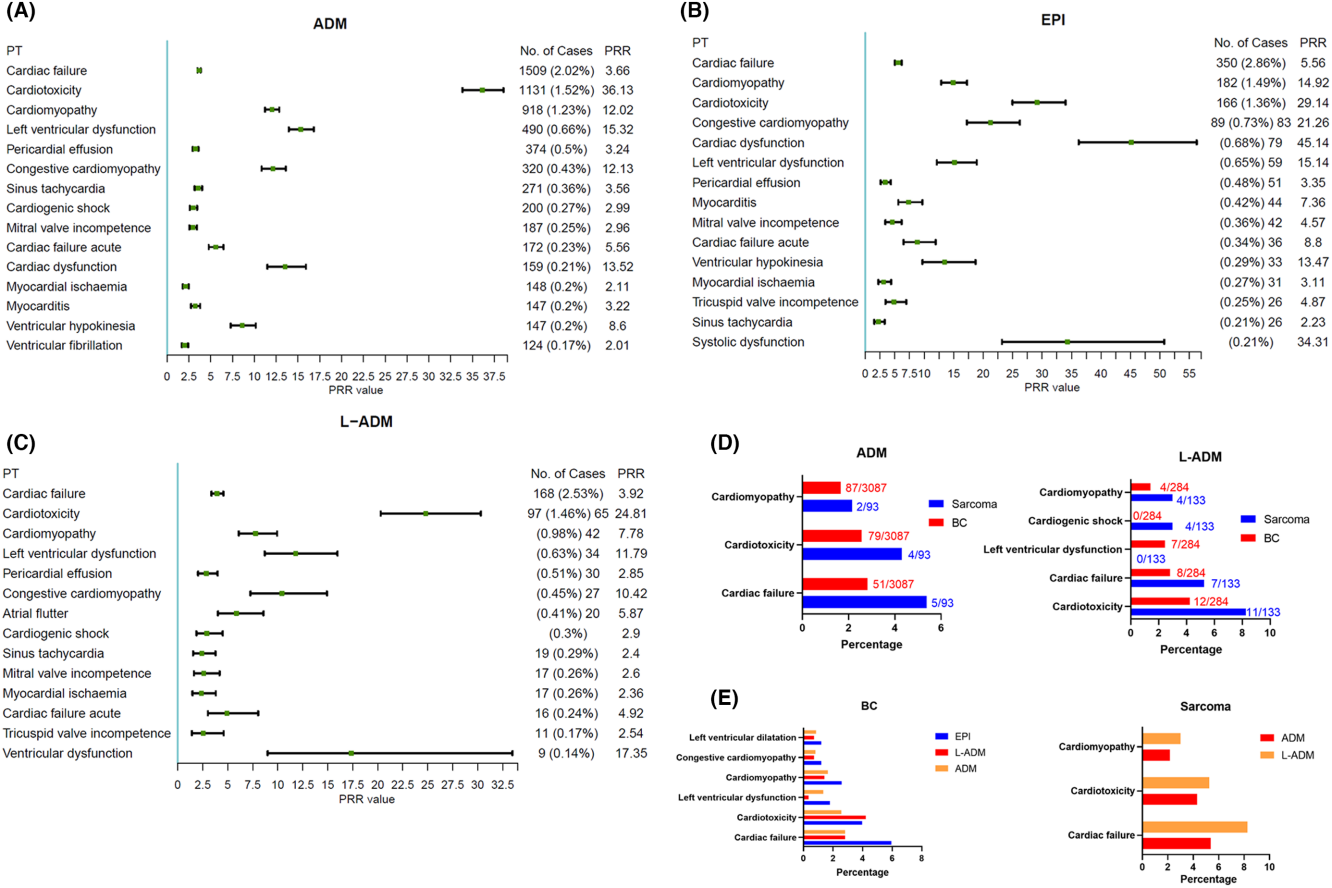
Frequency and population share of common cardiac disorders‐related adverse events (AEs) for three anthracyclines and comparison of the differences in sarcomas and breast cancer (BC). (A–C) The number, percentage, and specific PRR values of cardiac disorders‐related AEs were visualized. (D) The frequency and the percentage of common cardiac disorders reported with the same cancer type were compared separately.

The frequency, frequency population percentage, and PRR values of AEs associated with cardiac disorders were compared for the three anthracyclines from the FAERS database. Cardiac failure is the most frequent AEs among ADM, EPI, and L‐ADM; nearly 2% of patients eventually develop cardiac failure (Figure 3A–C). The frequency was followed closely by cardiotoxicity and cardiomyopathy, and the reporting rate fluctuated by approximately 1–1.5%. The previous univariate logistic regression analysis based on MHRA signals suggested that BC and sarcomas have a higher probability of cardiac disorders. Therefore, the frequency and percentage of common AEs of ADM and L‐ADM in the two tumors were compared (EPI was excluded because it is rarely used in sarcomas). Figure 3D shows that patients with sarcomas exceeded BC in the probability of occurrence of almost all common AEs. These results demonstrated that patients with sarcomas receiving anthracycline‐based chemotherapy may be at higher chance for cardiotoxicity. To more fully assess cancer therapy‐related cardiac dysfunction (CTRCD) in sarcoma patients treated with anthracycline‐based systems, we executed a single‐center clinical retrospective study.

### 3.4 Cardiotoxicity protection of dexrazoxane in patients younger than 18 years of age

**FIGURE 4:**
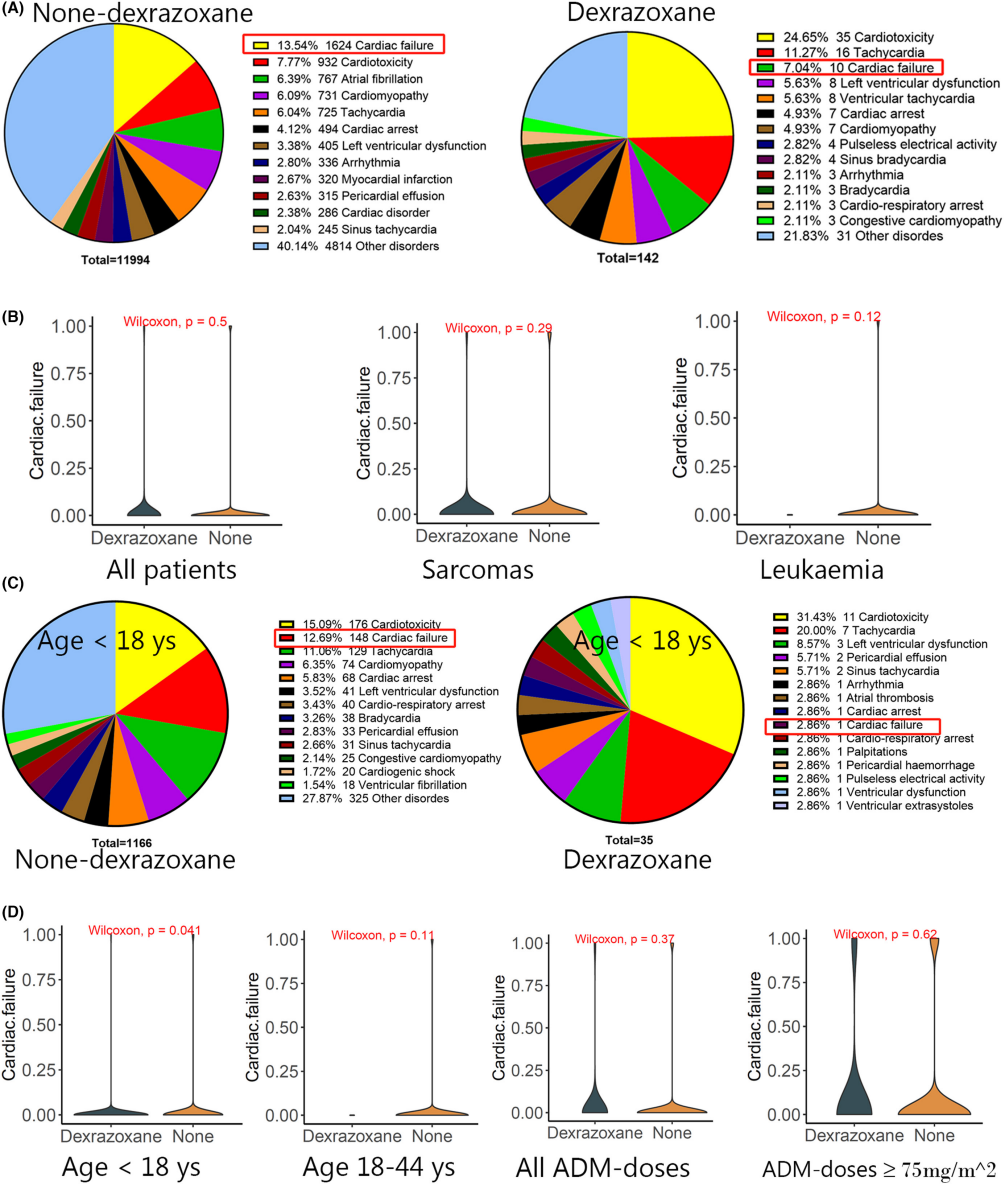
Comparison of the likelihood of cardiac failure between the dexrazoxane and non‐dexrazoxane groups. (A) Pie chart visualizing the percentage of different “preferred terms” in the number of all heart diseases between the dexrazoxane and non‐dexrazoxane groups. (B) The Wilcoxon test compares cardiac failure between the dexrazoxane and non‐dexrazoxane groups in all‐tumor species, sarcomas, or leukemias. (C) Pie chart visualizing the percentage of different “preferred terms” in the number of all heart diseases in adolescents with tumors. (D) The Wilcoxon test compares cardiac failure between the dexrazoxane and non‐dexrazoxane groups in different ages and ADM concentrations. *p* < 0.05 were considered statistically significant.

Those patients who had both anthracyclines and dexrazoxane/ZINECARD in the “DRUGNAME” were counted, and the results suggest that 717 patients were treated with dexrazoxane (Figure S2). Ninety‐seven percent of these patients with a history of dexrazoxane treatment were also treated with ADM. Therefore, we selected these patients treated with ADM plus dexrazoxane for statistical analysis. Forty‐one percent of the patients in the dexrazoxane group were under 18 years of age, while 11.0% of the patients in the non‐dexrazoxane group were in that age range (Table S2). In addition, uploads of AE reports in the dexrazoxane group were primarily from the United States and Canada. And perhaps sarcoma patients are more likely to be treated with dexrazoxane. Statistical analysis revealed that the number of cardiac failures as a percentage of all uploaded heart disorder reports decreased from 13.5% in the non‐dexrazoxane group to 7.0% in the dexrazoxane group (Figure 4A). Nonetheless, there was no significant difference in the likelihood of cardiac failure between the two groups with and without dexrazoxane treatment, either for all tumor types or when evaluating sarcomas and leukemias alone, where dexrazoxane was most used (Figure 4B). However, for those patients younger than 18 years of age treated with ADM, the number of cardiac failures as a proportion of all reported events of heart disorders was 12.7% in the non‐dexrazoxane group and only 2.9% in the dexrazoxane group (Figure 4C). Moreover, treatment with dexrazoxane may significantly reduce the likelihood of cardiac failure in oncology patients younger than 18 years of age (Figure 4D). No significant differences in the likelihood of heart failure between the non‐dexrazoxane and dexrazoxane groups could be found for other age groups and different ADM concentrations (Figure 4D). These results suggest that adolescent oncology patients are more likely to benefit from dexrazoxane prevention of cardiac failure.

### 3.5 The basic clinical features of patients with BSTSs originating at our hospital

**FIGURE 5:**
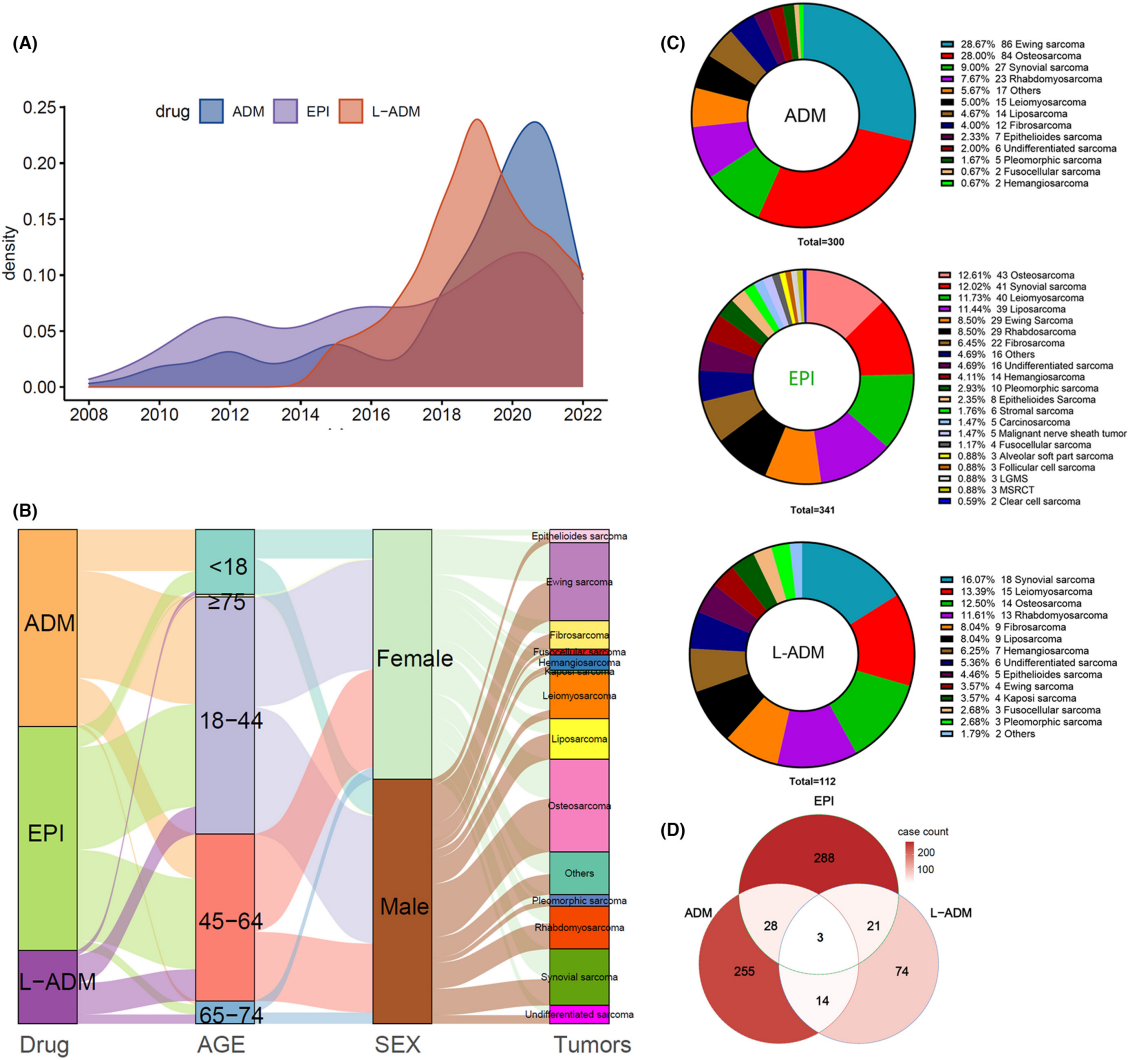
Distribution of essential clinical characteristics of patients receiving anthracycline‐containing chemotherapy from our single center. (A) Density plot of the number of patients with STBSs who received anthracycline‐containing therapy. (B) Mulberry map of clinical characteristics of patients with ADM, EPI, and L‐ADM. (C) The number and percentage of different tumor types in patients treated with anthracyclines were counted separately. (D) Overlapping Wayne plots of individual identities of patients treated with three medications based on the unique identification ID of patients.

Unlike the FAERS database, the number of patients with BSTSs receiving chemotherapy with EPI‐containing regimens (341 cases) at our center exceeded that of patients receiving chemotherapy with ADM‐containing regimens (300 cases). Since 2008, patients diagnosed with BSTSs have been treated at our institution with a chemotherapy regimen containing ADM (one patient) or EPI (one patient), and five patients who received L‐ADM appeared in 2015 (Figure 5A). Over time, the number of patients with ADM and L‐ADM applications has shown a relatively fast growth rate, while the growth trend of EPI applications has been relatively slow. Overall, the proportion of men and women with BSTSs who received anthracycline‐based chemotherapy was very similar, and there were no significant differences between the three drugs (Figure 5B, Table S1). The age distribution at the overall level was highest in the 18–44 years old group, followed by the 45–64 years old group and the <18 years old group, and 61% of BSTSs patients initially treated with anthracycline‐based chemotherapy were not older than 44 years (Table S1). Seventy‐five percent of patients receiving ADM were younger than 45, a higher percentage than EPI (54.8%) and L‐ADM (43.4%). Conversely, a relatively higher percentage of patients treated with EPI or L‐ADM were older than 45. In addition, the top four tumor frequencies for which ADM‐containing chemotherapy regimens were applied were Ewing sarcoma, osteosarcoma, synovial sarcoma, and rhabdomyosarcoma, whereas the top four tumors for EPI were osteosarcoma, synovial sarcoma, leiomyosarcoma, and liposarcoma (Figure 5C). Synovial sarcoma and leiomyosarcoma were the most common BSTSs in the L‐ADM treatment group.

Several factors, such as side effects of chemotherapy, patients' history of chemotherapy in other hospitals before admission, new drug launches, and adjustments in treatment regimens, have led to a proportion of patients receiving more than two anthracycline‐based systemic chemotherapy treatments (Figure 5D). Of these, 33.9% of patients treated with L‐ADM also had a history of treatment with ADM or/and EPI.

### 3.6 Evaluation of cardiotoxicity‐related markers in patients with BSTSs

**FIGURE 6:**
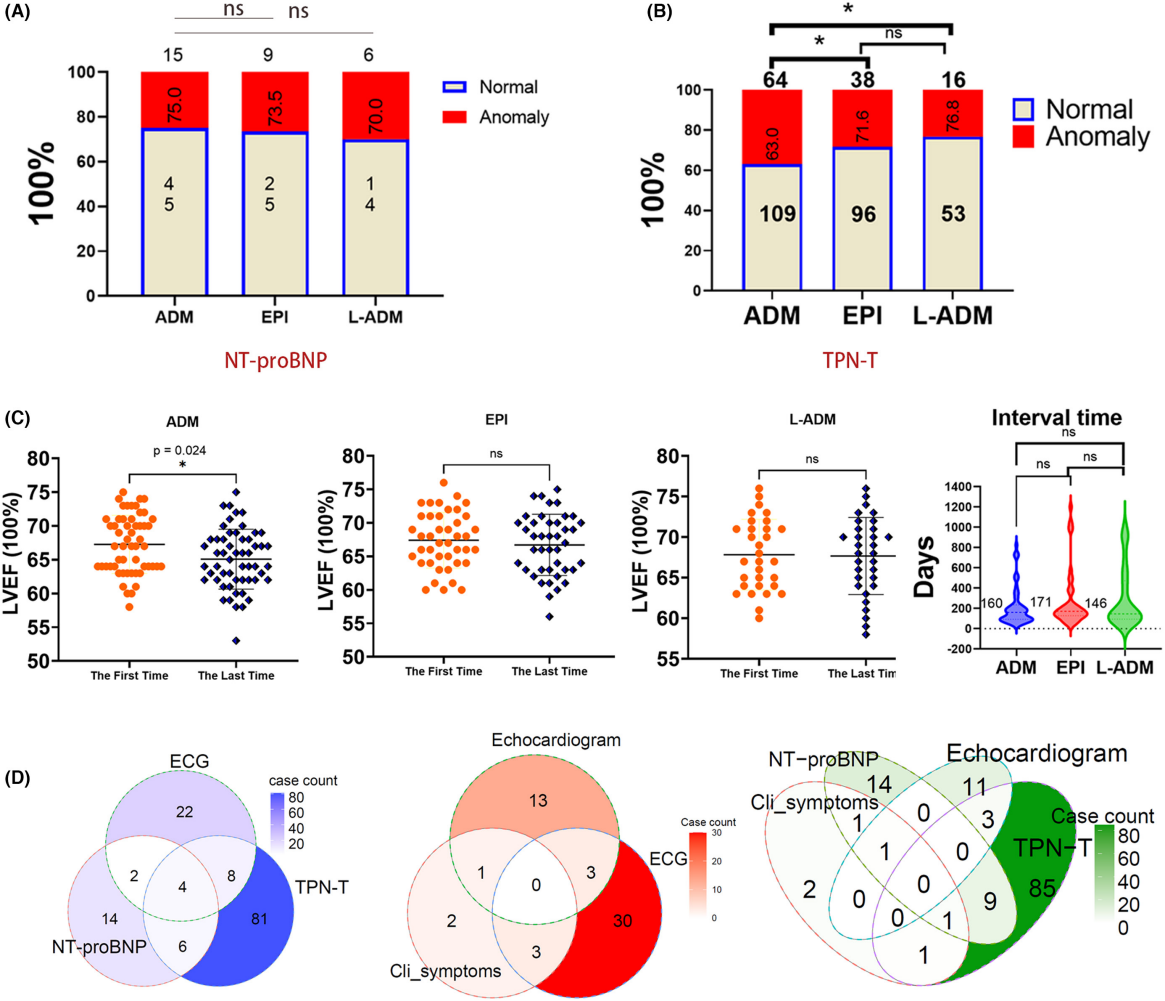
Evaluation of cardiotoxicity‐related markers in patients with STBSs at our center. (A, B) Comparison of differences between different drug groups that developed abnormal NT‐ProBNP or TPN‐T values after receiving anthracycline‐containing chemotherapy. The numbers in the blue and red boxes represent the number of patients with normal serum NT‐ProBNP or TPN‐T values, and the numbers in the red boxes represent the number of patients with abnormal serum NT‐ProBNP or TPN‐T values. (C) In the left ventricular ejection fraction (LVEF) assay, the difference between the first‐measurement baseline values compared to the last‐measurement values (the value with the largest decrease in LVEF was selected if three or more detection event points existed) was compared. (D) Overlapping Wayne plots of abnormal markers based on unique identification ID of patients.

NT‐ProBNP values were collected from patients with BSTSs treated with anthracycline‐containing chemotherapy regimens and screened for those who had been tested at least twice. Abnormal NT‐ProBNP values were determined as follows: men (≤50 years old, ProBNP ≥89 ng/L; >50 years old, ProBNP ≥228 ng/L), women (≤50 years old, ProBNP ≥154 ng/L; >50 years old, ProBNP >335 ng/L). The results suggested that 25% of patients treated with ADM experienced abnormal BNP values, slightly lower than EPI (27.5%) and L‐ADM (30%) but not significantly different (Figure 6A). However, 37% of patients treated with ADM had abnormal serum TPN‐T concentrations (abnormal TPN‐T values: >14.0 ng/L), which occurred with a significantly higher probability than EPI (28.4%) and L‐ADM (23.2%). However, there was no significant difference between EPI and L‐ADM (Figure 6B). Subsequently, LVEF values based on echocardiographic detection that spanned at least one cycle of an anthracycline‐containing chemotherapy regimen were counted. Only patients treated with ADM had final LVEF values significantly lower than their first detected values (Figure 6C). In none of the drug groups; however, did the LVEF fall below 50%. In addition, there was no significant difference in the period between measurements of echocardiograms for any of the three drugs (ADM: 160 days; EPI: 171 days; L‐ADM: 146 days, *p* > 0.05). On the other hand, the number of patients in each drug group who experienced a decrease in LVEF of more than 10% from baseline was also counted. The percentage of abnormalities in ADM, EPI, and L‐ADM was 20% (11/55), 9.8% (4/41), and 9.7% (3/31), respectively. ADM is more likely to induce a decrease in LVEF than the other two drugs.

In addition, the numbers of patients who developed markedly abnormal electrocardiograms and six patients who required adjustment of the chemotherapy regimens due to symptoms or signs of cardiotoxicity were statistically analyzed. Of note, the six patients were all receiving ADM‐containing chemotherapy regimens. We integrated all the different cardiotoxicity‐related markers in the three drugs and then performed intersection analysis based on Wayne plots. As shown in Figure 6D, there were no perfectly overlapping markers, and the only ones with ≥50% overlap were clinical symptoms and NT‐ProBNP abnormalities (50%, 3/6). We found that only 5.9% (1/17) of those patients who developed a 10% decrease from baseline in LVEF developed clinical symptoms, and the percentage was even lower in the TPN‐T abnormal group (2.0%, 2/99). These results suggest that most of BSTSs patients receiving anthracycline‐containing chemotherapy may exhibit asymptomatic cardiotoxicity.

Combining all evaluation metrics, we ultimately identified only two patients with symptomatic CTRCD based on the 2022 ESC guidelines.^33^ However, none of the patients included in the analysis experienced permanent termination of antitumor therapy or death during follow‐up.

## 4 DISCUSSION

In this study, based on data from the FAERS database, we found that patients with sarcomas were more likely than patients with other malignancies to experience AEs related to heart disorders after receiving chemotherapy with anthracycline‐containing regimens. However, data derived from FAERS tend to be missing precise measurements of echocardiography, electrocardiogram, serum troponin‐T, N‐terminal BNP, etc., to more objectively assess cardiotoxicity induced by anthracycline‐containing chemotherapy regimens. Therefore, a combined analysis of the data from the two components may provide a more comprehensive picture of the cardiotoxicity of anthracyclines in patients with sarcomas. The results from the FAERS database demonstrate the preference of the three drugs in terms of age, sex, and tumor type of patients in previous clinical use. For example, the percentage of ADM (11.0%) application in the age group of less than 18 years was significantly more than that of EPI (2.0%) and L‐ADM (3.5%). Nevertheless, the types and probabilities of occurrence of high‐frequency AEs associated with these three drugs were very similar. The nearly 2.5% reporting rate of cardiac failure is also comparable to previous studies.^34^ In addition, our study suggests that adolescents are more likely to experience cardiac disorders overall than patients in other age groups. The reason may be that a significantly higher percentage of adolescent children were treated with ADM (93.5%, 8379 patients) than with EPI or L‐ADM. And cardiotoxicity was more likely to occur in the ADM group than in the EPI and L‐ADM groups, based on our study. However, our study also failed to provide a definitive answer regarding the need for additional cardiac function monitoring in CCS.^35^

Although the FAERS database does not give the exact number of cycles receiving ADM‐based chemotherapy, we relied only on single‐cycle doses with clear values in the DOSE_AMT column. Even though, we could not determine whether the increased likelihood of cardiac disorders with ADM of ≥75 mg/ m^2^/cycle compared with lower cycle doses was attributable to the higher final cumulative dose or the high single‐cycle dose. A previous study suggested that the dose‐escalated group (EPI: 110 mg/m^2^ every 21 days) had a higher rate (15.7% vs. 7.3%) of asymptomatic cardiotoxicity and a lower median total cumulative dose with EPI‐containing chemotherapy compared to the dose‐dense group in BC treatment.^36^ Therefore, perhaps we need to give more attention to potential cardiotoxicity when using chemotherapy regimens with high cycle doses of ADM or EPI. Finally, our study reveals a higher probability of cardiotoxicity in BSTSs patients relative to other cancer types, which is less mentioned in previous studies.

Considering that anthracyclines are indispensable in the systemic treatment of many types of BSTSs, how to attenuate the cardiotoxicity of anthracyclines has become an important topic. We found that L‐ADM was less likely to develop drug‐related heart disorders than EPI and ADM in whole tumors (Figure 2B). Therefore, L‐ADM might function as an alternative to ADM for anti‐BSTSs. Currently, two L‐ADM drugs (Doxil and Lipo‐Dox®) have been approved for treating Kaposi's sarcoma.^37^ Doxil and Lipo‐Dox®, as members of nanomedicines, can achieve enhanced permeability and retention effects based on nano‐sized drug delivery systems (DDSs).^38^ DDSs can improve drug delivery and release within the tumor through passive or active targeting strategies, thereby achieving antitumor efficacy while attenuating chemotherapy side effects.^39^ As a result, more and more nanomedicines are being explored to treat BSTSs, and some good progress has been made at the preclinical and clinical levels.^37^, ^39^, ^40^ Two other nanomedicines, DaunoXome® and Liposomal mifamurtide (MEPACT), have also been approved for use in Kaposi's sarcoma and osteosarcoma, respectively.^37^ Docetaxel‐loaded mPEG‐PLA nanoparticles also demonstrated higher intratumoral drug concentration, tumor inhibition rate, and safety than conventional docetaxel injection.^41^ In addition, nanotechnology serves as a versatile mounting platform to achieve inhibition of BSTSs while assessing tumor morphology changes in real‐time by mounting cytotoxic drugs, photodynamic therapeutic materials, small interfering RNA, or in vivo imaging materials.^39^, ^42^, ^43^, ^44^ These results suggest that the DDS may represent a powerful strategy for improving therapeutic efficacy and reducing toxicity of chemotherapeutics in treatments of BSTSs.

Osteosarcoma, Ewing sarcoma, and synovial sarcoma remain the more common tumors currently receiving anthracycline‐based systemic chemotherapy at our center. In contrast to the clinical characteristics of the patients in the FAERS database, EPI was used at a significantly higher rate in our center (7.2% vs. 45.3%). On the one hand, multiple studies have concluded that EPIs and ADMs have comparable efficacy in controlling sarcomas.^45^, ^46^ On the other hand, we may recommend EPI‐containing regimens with adequate efficacy validation when considering patients with relatively severe cardiac disease or higher risk cardiotoxicity before chemotherapy.

Attributable to appropriate prechemotherapy assessment of cardiac functions, these anthracycline‐treated patients with BSTSs did not experience cardiotoxicity‐related deaths during chemotherapy in our center. Our study also reveals that ADM is more likely to trigger asymptomatic underlying cardiac function impairment than EPI and L‐ADM, based on serum TPN‐T abnormalities and decreased LVEF. Although an increasing number of cardiotoxicity‐related markers have been proposed, the intersection between these markers is not as high as we thought. Finding a single marker abnormality may be difficult to determine whether chemotherapy can be continued with the original anthracycline‐containing regimen, especially for patients with asymptomatic CTRCD. Most patients who present with mild abnormalities in blood biochemistry, ECG, or echocardiography often have to remain on their original regimen because of the scarcity of effective non‐anthracycline‐based chemotherapeutic regimens for many subtypes of BSTSs. However, we found that cardiotoxicity‐related markers in these patients gradually returned to normal levels after completion of chemotherapy.

Limitations: We cannot avoid some of the inherent shortcomings of the FAERS database. Due to inherent limitations and reporting biases, data from spontaneous reporting systems and associated disproportionality analyses cannot be used to infer causality and provide incidence and risk assessments. Although the data from multivariate logistic regression and disproportionality analyses suggested significant differences, all of our conclusions speculate on the probabilistic influence of some factors on the occurrence of cardiac disorders. To validate the FAERS analysis results and better reveal the differences between anthracyclines on cardiac function in patients with BSTSs, we executed a single‐center retrospective study. In addition, it is a pity that echocardiography at our center neglected to detect global longitudinal strain (GLS) values, which poses a challenge in screening for mild asymptomatic CTRCD (the LVEF of all the patients was higher than 50%, so severe and moderate grades were excluded).

In conclusion, based on data collected from the FAERS database and a single‐center retrospective study, our study suggests that patients receiving anthracycline‐containing chemotherapy regimens with SBTSs are more likely to develop cardiac disorder‐associated AEs than other tumors. In addition, ADM significantly increases the risk of TPN‐T abnormalities and a downward trend in LVEF compared with EPI and L‐ADM and increases the odds of interrupting the original regimens due to cardiotoxicity.

## AUTHOR CONTRIBUTIONS

**Zeming Mo:** Conceptualization (equal); data curation (equal); formal analysis (equal); investigation (equal); methodology (equal); resources (equal); software (equal); validation (equal); visualization (equal); writing – original draft (equal). **Yaotiao Deng:** Conceptualization (equal); data curation (equal); writing – original draft (equal). **Yiwen Bao:** Conceptualization (equal); data curation (equal); formal analysis (equal); methodology (equal); software (equal); writing – original draft (equal). **Jie Liu:** Conceptualization (equal); resources (equal); writing – original draft (equal). **Yu Jiang:** Conceptualization (equal); data curation (equal); funding acquisition (lead); project administration (equal); supervision (equal); writing – review and editing (equal).

## FUNDING INFORMATION

This work was supported by the Medical science and technology project of Sichuan Provincial Health Commission (21PJ005).

## CONFLICT OF INTEREST STATEMENT

The authors declare no conflict of interest.

## ETHICAL STATEMENT

This study was performed in line with the principles of the Declaration of Helsinki. Approval was granted by the Ethics Committee of West China Hospital of Sichuan University, China (No. 627 in 2020). The requirement for individual informed consent was waived.

## Supporting information


Figure S1.

Figure S2.

Table S1.

Table S2.

## Data Availability

These FEARS data were derived from the following resources available in the public domain: The FDA Adverse Event Reporting System (https://fis.fda.gov/extensions/FPD‐QDE‐FAERS/FPD‐QDE‐FAERS.html.) The other data supporting this study's findings are available in this article's supplementary material.
