# Supplementary figures and images for: Evaluation of cardiotoxicity of anthracycline‐containing chemotherapy regimens in patients with bone and soft tissue sarcomas: A study of the FDA adverse event reporting system joint single‐center real‐world experience
Source: Cancer Med. 2023 Dec 6;12(24):21709–24. doi: 10.1002/cam4.6730 (PMC10757145; doi:10.1002/cam4.6730)

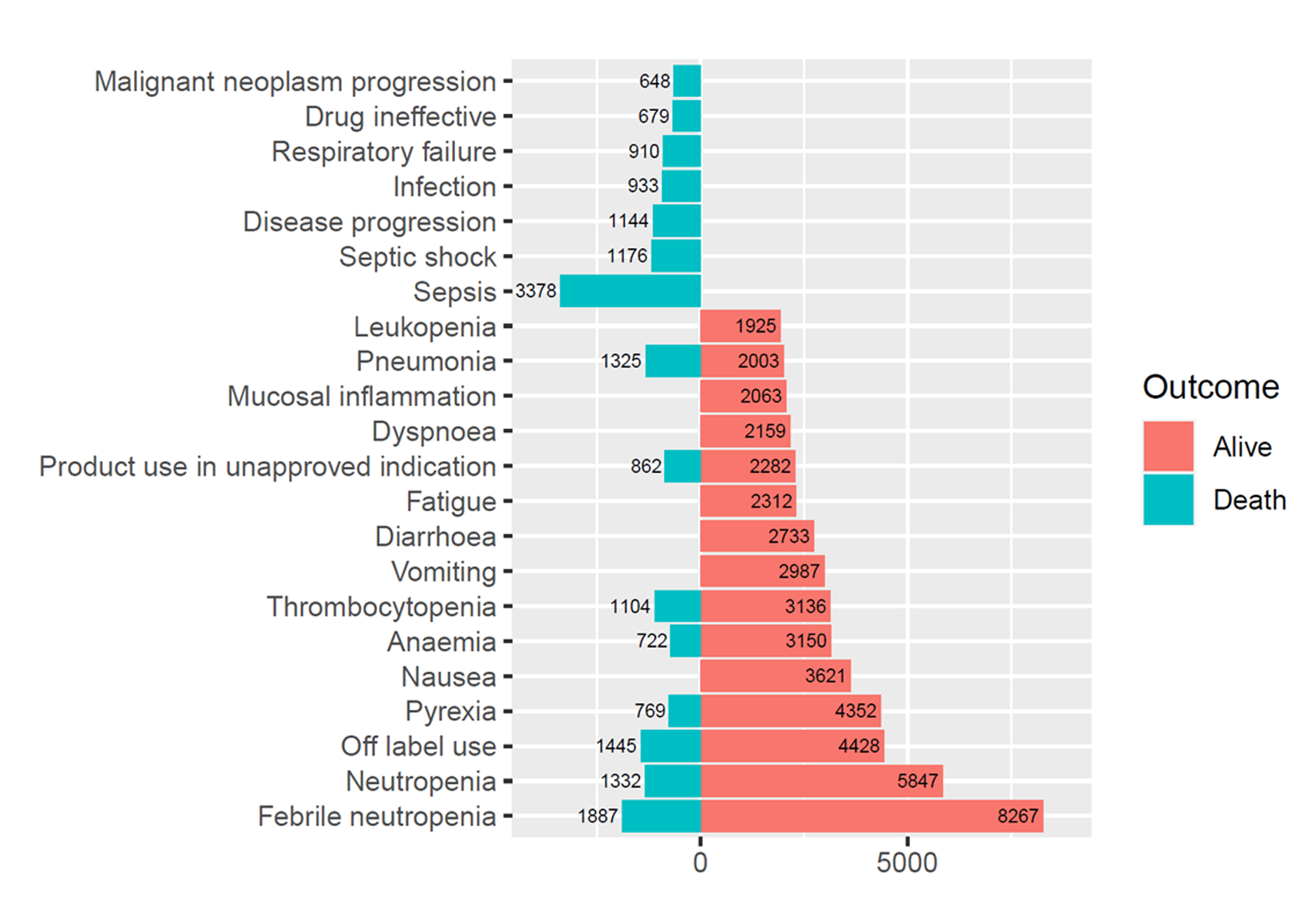

Supplement: Supplementary file 1 — Figure S1. [file CAM4-12-21709-s003.tif]

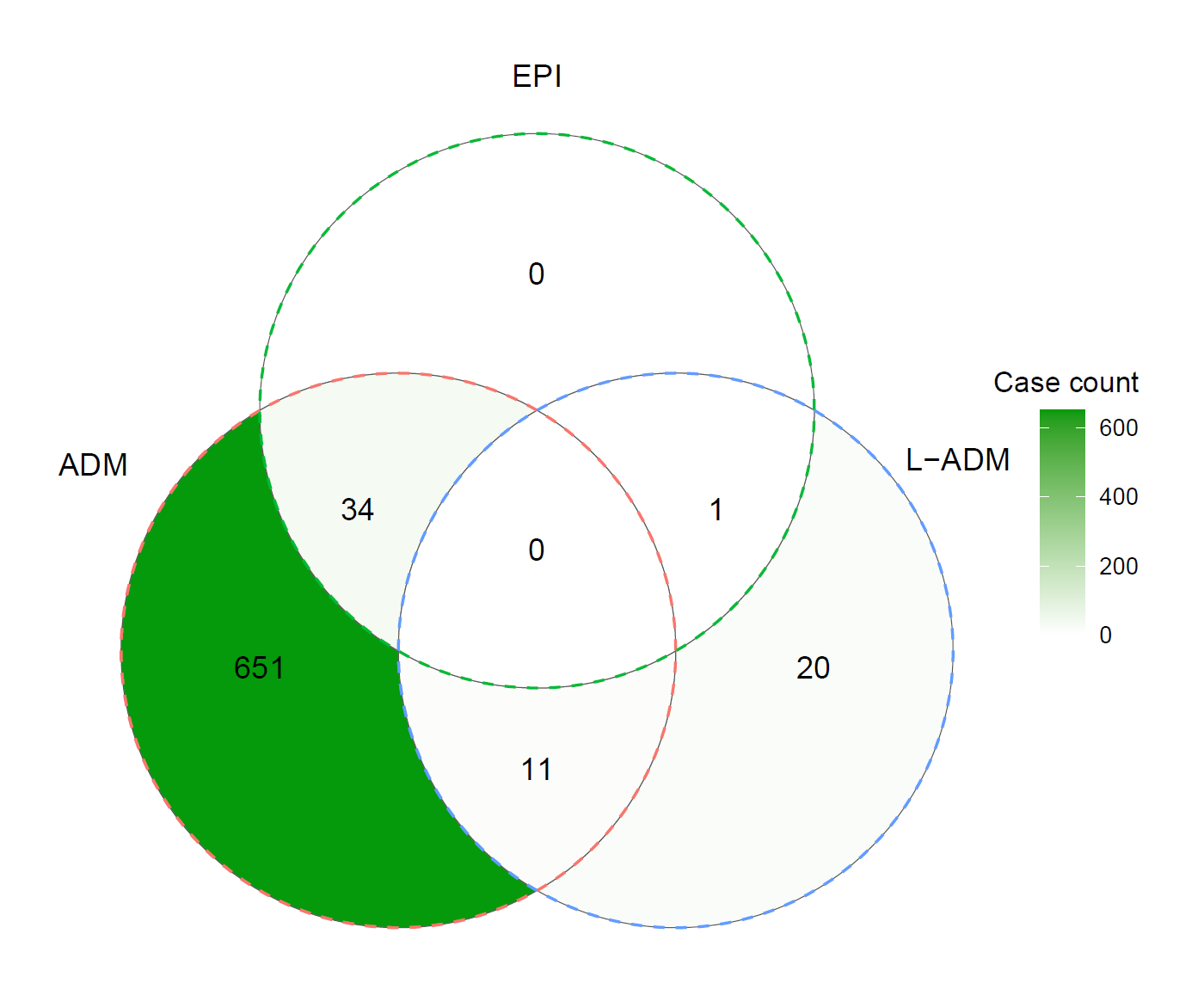

Supplement: Supplementary file 2 — Figure S2. [file CAM4-12-21709-s004.tif]
