# Supplementary material for: Evaluation of cardiotoxicity of anthracycline‐containing chemotherapy regimens in patients with bone and soft tissue sarcomas: A study of the FDA adverse event reporting system joint single‐center real‐world experience
Source: Cancer Med. 2023 Dec 6;12(24):21709–24. doi: 10.1002/cam4.6730 (PMC10757145; doi:10.1002/cam4.6730)
Supplement: Supplementary file 3 — Table S1. [file CAM4-12-21709-s002.docx]

Supplementary table 1. The basic clinical features of patients with bone and soft tissue sarcomas at our center

| **Characteristic** | **ADM**, N = 300^1^ | **EPI**, N = 341^1^ | **L-ADM**, N = 112^1^ | **p-value**^2^ |
| --- | --- | --- | --- | --- |
| AGE |  |  |  | <0.001 |
| <18 | 63 (21%) | 30 (8.8%) | 6 (5.4%) |  |
| 18-44 | 163 (54%) | 156 (46%) | 42 (38%) |  |
| 45-64 | 68 (23%) | 138 (40%) | 48 (43%) |  |
| ≥65 | 6 (2.0%) | 17 (5.0%) | 16 (14%) |  |
| GENDER |  |  |  | 0.5 |
| Female | 146 (49%) | 172 (50%) | 62 (55%) |  |
| Male | 154 (51%) | 169 (50%) | 50 (45%) |  |
| Tumor_types |  |  |  |  |
| Epithelioides sarcoma | 7 (2.3%) | 8 (2.3%) | 5 (4.5%) |  |
| Ewing sarcoma | 86 (29%) | 29 (8.5%) | 4 (3.6%) |  |
| Fibrosarcoma | 12 (4.0%) | 22 (6.5%) | 9 (8.0%) |  |
| Fusocellular sarcoma | 2 (0.7%) | 4 (1.2%) | 3 (2.7%) |  |
| Hemangiosarcoma | 2 (0.7%) | 14 (4.1%) | 7 (6.2%) |  |
| Kaposi sarcoma | 0 (0%) | 0 (0%) | 4 (3.6%) |  |
| Leiomyosarcoma | 15 (5.0%) | 40 (12%) | 15 (13%) |  |
| Liposarcoma | 14 (4.7%) | 39 (11%) | 9 (8.0%) |  |
| Osteosarcoma | 84 (28%) | 43 (13%) | 14 (12%) |  |
| Others | 17 (5.7%) | 46 (13%) | 2 (1.8%) |  |
| Pleomorphic sarcoma | 5 (1.7%) | 10 (2.9%) | 3 (2.7%) |  |
| Rhabdomyosarcoma | 23 (7.7%) | 29 (8.5%) | 13 (12%) |  |
| Synovial sarcoma | 27 (9.0%) | 41 (12%) | 18 (16%) |  |
| Undifferentiated sarcoma | 6 (2.0%) | 16 (4.7%) | 6 (5.4%) |  |
| ^1^n (%) | | | | |
| ^2^Pearson's Chi-squared test | | | | |
