# Supplementary material for: Evaluation of cardiotoxicity of anthracycline‐containing chemotherapy regimens in patients with bone and soft tissue sarcomas: A study of the FDA adverse event reporting system joint single‐center real‐world experience
Source: Cancer Med. 2023 Dec 6;12(24):21709–24. doi: 10.1002/cam4.6730 (PMC10757145; doi:10.1002/cam4.6730)
Supplement: Supplementary file 4 — Table S2. [file CAM4-12-21709-s001.docx]

**Supplementary Table 2**

Basic clinical characteristics of patients with and without dexrazoxane in FAERS database.

| **Characteristic** | **Dexrazoxane**, N = 668^1^ | **None**, N = 77,133^1^ | **p-value**^2^ |
| --- | --- | --- | --- |
| AGE |  |  | <0.001 |
| <18 | 273 (41%) | 8,106 (11%) |  |
| >75 | 11 (1.6%) | 4,140 (5.4%) |  |
| 18-44 | 124 (19%) | 11,130 (14%) |  |
| 45-64 | 54 (8.1%) | 18,396 (24%) |  |
| 65-74 | 24 (3.6%) | 9,706 (13%) |  |
| Unknown | 182 (27%) | 25,655 (33%) |  |
| Country |  |  |  |
| CA | 163 (24.4%) | 4,482 (5.8%) |  |
| CN | 19 (2.8%) | 2,572 (3.3%) |  |
| US | 322 (48.2%) | 14,643 (18.9%) |  |
| Other Countries | 98 (14.7%) | 38565 (50%) |  |
| Unknown | 66 (9.9%) | 16,871 (21.9%) |  |
| GENDER |  |  | 0.004 |
| Female | 243 (36%) | 31,668 (41%) |  |
| Male | 276 (41%) | 27,198 (35%) |  |
| Unknown | 149 (22%) | 18,267 (24%) |  |
| INDI_PT |  |  | <0.001 |
| Breast cancer | 13 (1.9%) | 6,235 (8.1%) |  |
| Leukaemia | 118 (18%) | 11,612 (15%) |  |
| Lymphoma | 23 (3.4%) | 23,011 (30%) |  |
| Other diseases | 172 (26%) | 20,966 (27%) |  |
| Ovarian cancer | 1 (0.1%) | 1,522 (2.0%) |  |
| Sarcoma | 143 (21%) | 3,477 (4.5%) |  |
| Unknown | 198 (30%) | 10,310 (13%) |  |
| ^1^n (%) | | | |
| ^2^Pearson's Chi-squared test | | | |

Unknown indicates that there is no clearly labeled information.
